# Knowledge gaps in high-energy sacral fracture treatment and management^☆^

**DOI:** 10.1016/j.jcot.2026.103455

**Published:** 2026-04-30

**Authors:** Ashraf N. El Naga, Marko H. Neva, Ahmed Abdelazim Hassan, Gregory D. Schroeder, Mohammad El-Sharkawi, Sebastian F. Bigdon, Andrei F. Joaquim, Klaus Schnake, Richard Bransford, Emre Yilmaz

**Affiliations:** a Department of Orthopaedic Surgery, University of California San Francisco (UCSF), San Francisco, CA, USA; b Department of Orthopaedic and Trauma Surgery, Tampere University Hospital, Tampere, Finland and Tampere University, Finland; c Department of Orthopaedic and Trauma Surgery, Faculty of Medicine, Assiut University, Egypt; d Department of Orthopaedic Surgery, Rothman Institute at Thomas Jefferson University Hospital, Philadelphia, PA, USA; e Department of Traumatology and Orthopaedic Surgery, Inselspital University of Bern, Freiburgstrasse, 3010, Bern, Switzerland; f Neurosurgery Division, Department of Neurology, State University of Campinas, Campinas-Sao Paulo, Brazil; g Center for Spinal and Scoliosis Surgery, Malteser Waldkrankenhaus St. Marien, Rathsberger Strasse 57, 91054, Erlangen, Germany; h Department of Orthopedics and Traumatology, Paracelsus Private Medical University Nuremberg, Nuremberg, Germany; i Harborview Medical Center, University of Washington, Seattle, WA, USA; j Department of General and Trauma Surgery, BG University Hospital Bergmannsheil Bochum, Germany

**Keywords:** Sacral fractures, Pelvic injury, Spine trauma, Knowledge gaps, Treatment, Management

## Abstract

High-energy sacral fractures (HESF) are often challenging injuries due to the location of the sacrum at the junction of the spine and pelvis, the complex biomechanics of the pelvic ring and the wide spectrum of injury patterns, which make treatment standardization challenging. Despite advances in classification systems – particularly the AOSpine Sacral Injury Classification – significant gaps remain in understanding optimal management.

To review and discuss the management and identify knowledge gaps related to HESF.

A narrative review of the literature was conducted, focusing on five domains: 1. Relative instability and biomechanics affected in different injury patterns; 2. The significance of concomitant pelvic ring disruption; 3. Reduction goals and options; 4. Optimal instrumentation construct for specific injuries and 5. The role and timing of neural element decompression.

The evidence is limited by retrospective studies and heterogeneity. The instability criteria are poorly defined. The role of anterior pelvic fixation remains controversial, with conflicting evidence considering posterior-only versus combined approaches. Reduction goals lack consensus as well. Considering instrumentation techniques, different options were proposed, such as sacroiliac fixation, spinopelvic constructions, and triangular osteosynthesis – with insufficient data for comparison. Finally, evidence for guiding neural decompression is sparse and inconsistent.

The management of HESF is extremely based on expert opinion, despite advancements in the classification system. Further prospective, multicenter, and well-designed trials are strongly necessary to define better treatment algorithms and answer the raised knowledge gaps.

## 1 Introduction

Sacral fractures represent a unique entity for both pelvic and spine surgeons. The heterogeneity of these relatively rare fractures ranges from stable transverse sacral fractures below the sacroiliac (SI) joint to highly unstable pelvic ring injuries or spinopelvic dissociations, which can be very difficult to reduce and stabilize. While osteoporotic sacral fractures mainly result from ground-level falls, non-osteoporotic sacral fractures result from high-energy trauma, and are often associated with multiple injuries.^1^^,^^2^ While occurring as isolated injuries only 5% of the time, sacral fractures are part of a concomitant pelvic ring injury in 45% of patients.^3^ Correct diagnosis and optimal treatment require a thorough understanding of the injury morphology, lumbosacral biomechanics, salient injury considerations, and available treatment options when treating high-energy trauma patients.^4^ However, the relatively low incidence of sacral fractures coupled with the wide heterogeneity of patterns has greatly limited the available literature, with most current recommendations stemming predominantly from expert opinion.^5^

**Fig. 1:**
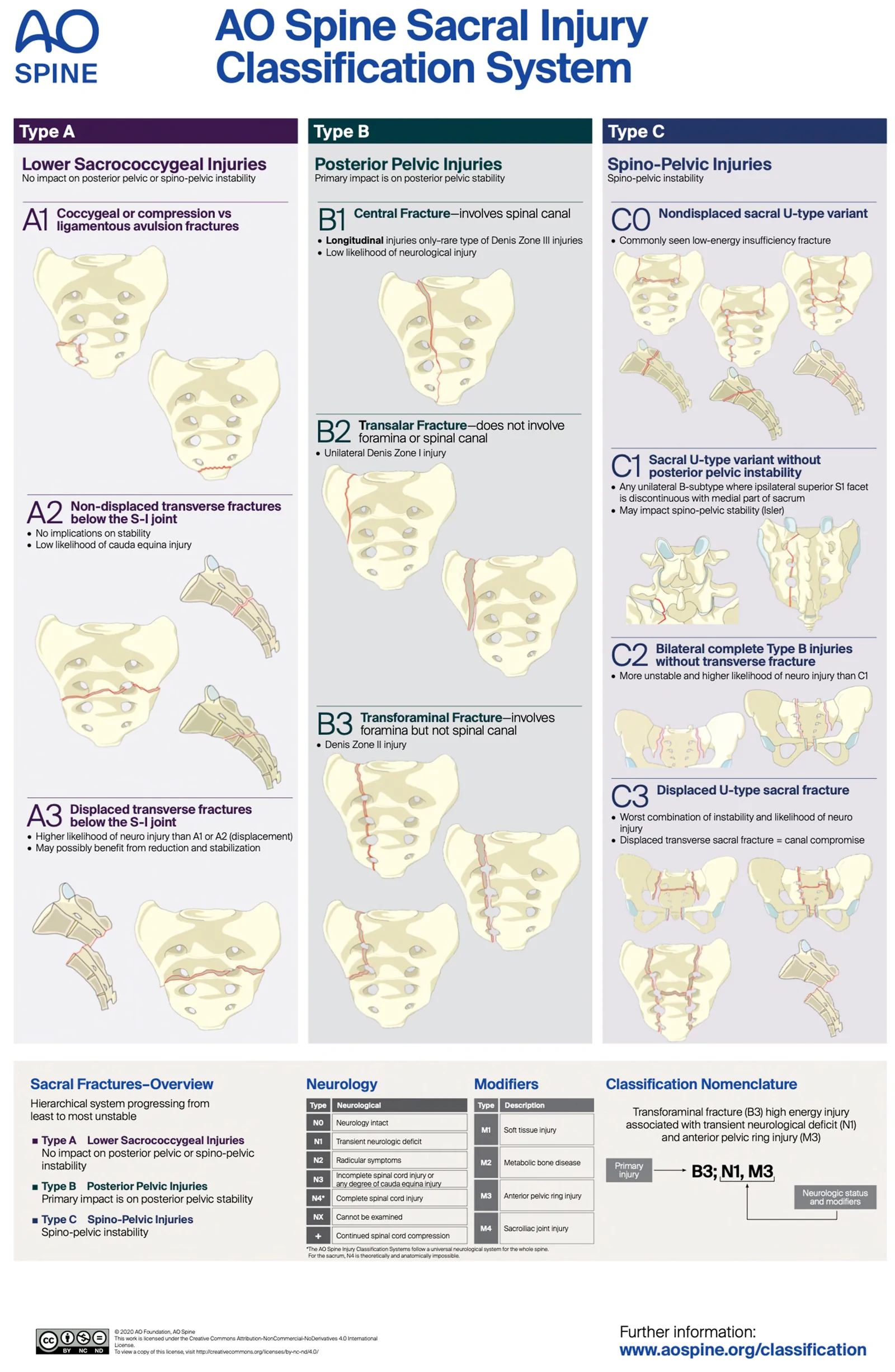
**AOSpine Sacral Injury Classification** Downloaded from: https://www.aofoundation.org/spine/clinical-library-and-tools/aospine-classification-systems"© AO Foundation, AO Spine, Switzerland".

The most recent advancement in the field of sacral injuries has been the consolidation of many prior injury classification systems into the AOSpine Sacral Injury Classification System (Fig. 1), which comprehensively categorizes injuries based on morphology in a rational hierarchical manner.^6^^,^^7^ While this classification system may at first seem less straightforward than earlier classification systems, Vaccaro et al. (2020) reported a substantial overall interrater agreement (κ = 0.75) for the AO Sacral Injury Classification, with the highest agreement for type A fractures (κ = 0.95) and the lowest for type C fractures (κ = 0.70).^8^ Now, with a widely accepted and comprehensive sacral injury classification system, we can evaluate the literature to guide treatment strategies for specific patients and injury subtypes.

Even though classifications have improved the understanding of different categories of sacral fracture, significant knowledge gaps remain. Therefore, the aim of this review is to critically evaluate the knowledge gaps in the management of high-energy sacral fractures.

## 2 Methods

From February to August 2025, we performed a manual search of the PubMed database for studies evaluating the management of HESF. Included studies were those addressing the biomechanics and management of HESF. Study selection was guided by the following knowledge gaps (Table 1):

1. Relative instability and biomechanics affected in different injury patterns
2. The significance of concomitant pelvic ring disruption
3. Reduction goals and options
4. Optimal instrumentation construct for specific injuries and
5. The role and timing of neural element decompression.

**Table 1:** Key domains in the management of sacral fractures, including assessment of stability, pelvic ring considerations, neural decompression strategies, reduction goals, and spinopelvic instrumentation. Each domain highlights current controversies and knowledge gaps regarding diagnosis, surgical decision-making and clinical outcomes.

Domain	Key Knowledge Gaps
Assessment of Stability	• Defining radiographic criteria of unstable injuries • Impact of the morphology L5/S1 facet disruptions on stability • Comparative rates of instability and consequential late malunion between injury patterns • Differences between pelvic and spine surgeons in the assessment of sacral fractures • Regional differences in the assignment of sacral fractures to spine or pelvic surgeons and in the treatment protocols
Pelvic ring considerations	• Understanding the relative contributions and clinical outcomes of anterior ring fixation in specific sacral fracture injury patterns
Neural Element Decompression	• Timing of decompression • Role of indirect versus direct decompression • Influence of primary neurologic impairment for the final outcome
Reduction Goals	• Quantifying acceptable residual sacral kyphosis in transverse patterns and long-term clinical outcomes
Spinopelvic Instrumentation Constructs	• Determining the optimal cranial extent of spinopelvic constructs based on injury pattern • Clinical outcomes of open versus percutaneous spinopelvic techniques • Role of supplemental spinopelvic fixation to facilitate weightbearing in B type injuries • Determining the absolute and relative indications of lumbosacral fusion • Effect of navigation for the accuracy of screw placement, surgical time and final outcome

Key domains in the management of sacral fractures, including assessment of stability, pelvic ring considerations, neural decompression strategies, reduction goals, and spinopelvic instrumentation. Each domain highlights current controversies and knowledge gaps regarding diagnosis, surgical decision-making and clinical outcomes.

These knowledge gaps were proposed based on regular meetings of the AOSpine Knowledge Forum Trauma and Infection, a study group supported by AO Spine, focused on the development of knowledge in spinal trauma. After that, a discussion based on experts' insights was used to propose future directions and personal experiences in the evaluated gaps.

## 3 Results

The results were presented and grouped according to one of the five questions raised in Methods.

### 3.1 Biomechanics and instability in high energy sacral injuries

The sacrum is the keystone of the pelvic ring, anchoring the spine to the pelvis and transferring biomechanical forces from the spine to the bilateral ilia and the lower extremities. The pelvis's ability to withstand weight-bearing is directly related to the degree of consequential instability in sacral and pelvic injuries. Sacral fractures occur along zones of weakness. The most common high-energy fracture mechanism is a vertically oriented fracture line after a fall from a height or motor vehicle collision, often resulting in a fracture through the S1 and S2 foramina.^9^^,^^10^ When exposed to excessive loads as a result of trauma, stress in the foramen can increase threefold.^11^ Further, relative to the rest of the posterior pelvis, the sacral ala often fractures due to comparatively lower bone mineral density.^12^

Sacral fractures often occur due to complex high-energy trauma resulting in a combination of direct axial compression, hemipelvis internal/external rotation, hemipelvis flexion/extension, vertical and anteroposterior translation.^10^^,^^13^

With regards to sacral injuries with a transverse component, direct compression force against the coccyx may lead to lower transverse sacral fractures, while upper transverse sacral fractures are often the result of a fall from a great height and axial load.^14^

Though not completely understood, a patient's overall spinal alignment, especially the lumbar lordosis, pelvic tilt, and sacral slope parameters, and a patient's position at the time of injury, might play a significant role in a patient's predisposition to injury with greater sagittal shear forces across the sacrum in patients with higher sacral slope 14, 15, 16.

Defining the degree of instability of sacral fractures remains a challenge to this day. Though instability has been commonly presumed if there is fracture displacement of 1cm or more^15^ there has been no study to formally quantify this, nor guidance as to whether such displacement is in reference to the anterior or posterior pelvic ring. Apart from the pelvic ring classification systems and prior to the AOSpine classification system, the most commonly used historical classifications regarding sacral fractures use different variables to define the degree of instability or injury severity including the Isler and Roy Camille classifications.^9^ While the Isler classification categorizes potential lumbosacral instabilities in vertically oriented fractures based on the morphology in relation to the L5/S1 facet joint,^17^ there is little data regarding the functional outcomes following these different patterns.^18^ For injuries with a transverse sacral component, the Roy-Camille classification categorizes injuries based on midsagittal kyphosis and translation, and beyond implications for fracture reduction, little is known as to comparative outcomes or reduction goals.

With regards to vertically oriented fracture lines, Lee et al. (2024) found that more surgeons recommended surgical stabilization for B1-type fractures (longitudinal sacral fracture medial to the foramen and involving the spinal canal) compared to B2-type fractures (longitudinal sacral fracture lateral to the foramen) (20.6% vs. 16.8%).^5^ This highlights that although the ordering of the B-type subclassification focused on neurological outcome, the degree of mechanical instability considered between injury subtypes may not correlate with the same hierarchical order. Additionally, Meissner-Haecker et al. (2022) interestingly observed only moderate agreement at the subtype level when comparing spine and pelvic trauma surgeons, highlighting potential areas of discordance based on training and experience.^19^

In conclusion, the ideal fracture classification should be comprehensive, logically structured, reliable, easy to use, clinically relevant, prognostically useful, and validated. The AO Sacral Injury Classification is well structured, captures nearly all injury patterns, proposing a crescent degree of severity from A to C, and is poised to serve as the basis for future investigations. However, there is a limited number of studies analyzing the etiology, fracture mechanisms, and relative degrees of instability between injury subtypes. A systematic analysis of the existing literature on the degree of instability and its defining variables, biomechanical testing of different fracture subtypes, and more methodologically robust clinical outcome studies are needed to fill this knowledge gap (Table 2).

**Table 2:** Comparison of commonly used sacral and pelvic fracture classification systems, outlining their underlying principles, strengths, and limitations. Systems are evaluated based on their consideration of fracture morphology, neurological involvement, mechanical stability, and pelvic ring integrity, as well as their clinical applicability and guidance for treatment decision-making. LC = Lateral Compression, APC = Anteroposterior Compression, VS = Vertical Shear, AO = Arbeitsgemeinschaft für Osteosynthesefragen, OTA = Orthopaedic Trauma Association

Classification	Pros	Cons
AO Sacral Injury Classification (6)	• based on fracture morphology, neurologic status, and case-specific modifiers • hierarchical order within overall type	• no specific treatment recommendations • may not reflect the hierarchal order with regards to mechanical instability
Denis (9)	• based on the fracture location with respect to the sacral foramina in order to predict the likelihood of neurological deficits	• not based on mechanical stability • does not include the anterior pelvic ring • only unilateral fractures included • no specific treatment recommendations
Roy-Camille (14)	• categorizes injuries based on midsagittal kyphosis and translation	• does not include the anterior pelvic ring • not hierarchical • no specific treatment recommendations
Isler (17)	• categorizes potential lumbosacral instabilities based on the fracture morphology in relation to the L5/S1 joint	• little existing prognostic value • no specific treatment recommendations
Tile (23)	• based on pelvic ring stability • clear hierarchical structure with increasing instability • indirectly incorporates the posterior ring (including the sacrum) • widely used and clinically intuitive	• limited detail regarding sacral fracture morphology • limited assessment of neurological involvement • no specific treatment recommendations • lumbosacral junction injuries are insufficiently addressed
Young Burgess (20)	• based on mechanism of injury (LC, APC, VS) • provides insight into force vectors and instability patterns • useful for initial assessment and trauma management • correlates with associated injuries and hemorrhage risk	• does not specifically classify sacral fractures • limited assessment of neurological involvement • moderate interobserver reliability • no specific treatment recommendations
AO/OTA (32)	• based on pelvic ring stability • clear hierarchical structure with increasing instability • more morphologic specificity • consideration of spinopelvic stability	• numerous subtypes • no inclusion of neurological deficit • less linkage to injury mechanism

Comparison of commonly used sacral and pelvic fracture classification systems, outlining their underlying principles, strengths, and limitations. Systems are evaluated based on their consideration of fracture morphology, neurological involvement, mechanical stability, and pelvic ring integrity, as well as their clinical applicability and guidance for treatment decision-making.

LC = Lateral Compression, APC = Anteroposterior Compression, VS = Vertical Shear, AO = Arbeitsgemeinschaft für Osteosynthesefragen, OTA = Orthopaedic Trauma Association.

### 3.2 Consideration of the anterior pelvic ring and the significance of ring disruptions

**Fig. 2:**
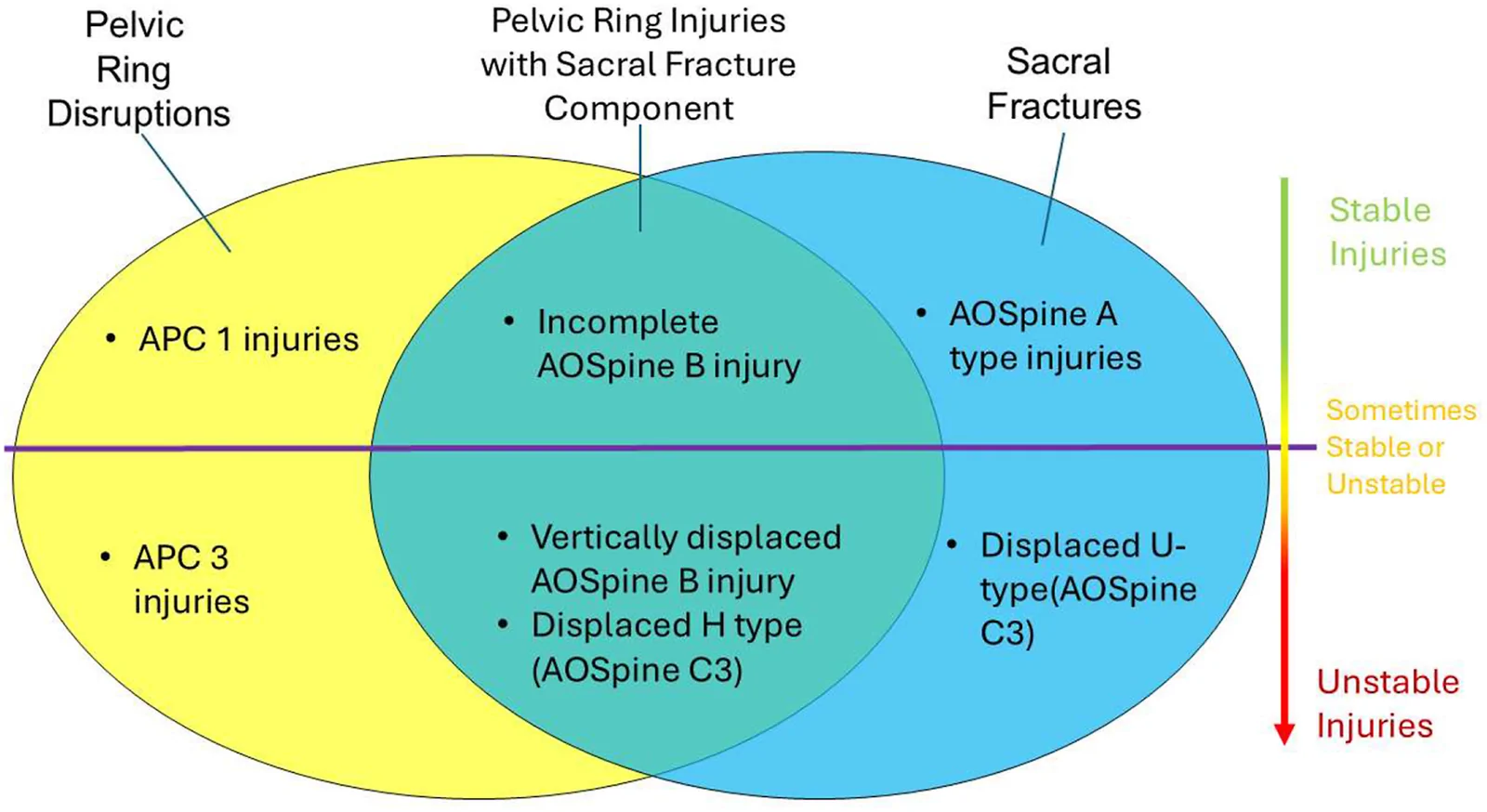
**Pelvic Ring Injuries and Sacral Fractures.** Pelvic ring injuries can be isolated or happen in conjunction with sacral fractures and vice versa with the existence of both stable and unstable injuries in isolated or combined injuries. Some examples of each scenario are listed in the diagram.

While the sacrum itself transmits load to the SI joints, the remainder of the pelvic ring functions as a fundamental biomechanical conduit for transmitting axial loads to the lower extremities. Contributing to the nuance of the discussion of pelvic ring injuries and sacral fractures is that while they often occur together, both stable and unstable injuries can present when one is absent (Fig. 2).

Sacral fractures in the presence of unstable pelvic ring injuries, further compromise structural integrity and load transmission.^9^^,^^20^ The configuration and severity of sacral involvement often serve as primary indicators of mechanical pelvic ring instability and often guide the selection of operative strategies,^21^ though anterior ring displacement is similarly very informative. While several sacral-specific classifications are discussed earlier, the two principal pelvic ring classification systems—**Tile** and **Young-Burgess**—are commonly used to stratify pelvic fractures and guide management.

The Tile classification stratifies pelvic ring injuries based on mechanical stability, particularly regarding the posterior pelvic arch. This system categorizes injuries into: Tile A—stable fractures with no posterior involvement; Tile B—rotationally unstable but vertically stable injuries with partial posterior ring disruption; and Tile C—fractures that are rotationally and vertically unstable due to complete loss of posterior pelvic ring integrity.^22^^,^^23^

In contrast, the Young-Burgess classification is mechanism-based, reflecting the direction of the traumatic force. It includes Anteroposterior Compression (APC) injuries, characterized by anterior ring diastasis; Lateral Compression (LC) injuries, which display variable anterior and posterior ring disruptions; Vertical Shear (VS) injuries, marked by cephalad displacement of the hemipelvis; and Combined Mechanism (CM) injuries that incorporate features of multiple force directions.^20^

One area that lacks complete clarity is identifying what constitutes sufficient fixation for specific fracture patterns when biomechanical studies naturally demonstrated increased stability with increased instrumentation and surgical invasiveness. For example, Sagi et al.^24^ in a cadaveric study demonstrated that adding a symphyseal plate to iliosacral screw fixation in APC III injuries leads to a significant reduction in both linear and rotational displacement, irrespective of the posterior construct used. Other studies concluded that adding anterior fixation in cases with transforaminal sacral fractures improved resistance to torsional loading.^25^ Additionally, Bodzay et al.^26^ in their finite element analysis study, they found that adding pubic rami screws not only stabilizes the anterior pelvic ring but also reduces motion across the sacral fracture site. Clinical studies have similarly demonstrated some disadvantages to more limited approaches in particular injuries. In their study on LC injuries, Tucker et al. concluded that although combined fixation was associated with prolonged operative time and blood loss, it was associated with less opioid use and earlier discharge from the hospital than isolated posterior fixation.^27^ This can be explained by the reduced incidence of anterior pelvic pain from pubic rami fractures.^28^ Other studies found that patients with posterior-only fixation for unstable pelvic injuries have a higher risk of fracture displacement, especially in patients with bilateral pubic rami fractures.^29^

However, on the other end of the spectrum, we find a multitude of studies that promote posterior-only fixation for pelvic ring injuries. Agrawal et al.^30^ recommended posterior-only fixation for LC fractures as it had comparable patient-reported outcomes to combined anteroposterior fixation with similar union rates and no loss of reduction. These findings were also supported by other studies^31^ that showed comparable functional outcomes and less operative time and blood loss in patients solely treated posteriorly, though Moussa et al.^31^ observed better intra-operative fracture reduction and increased infection in Tile C injuries treated with combined approaches.

The ongoing debate between isolated posterior fixation and combined anterior–posterior fixation goes beyond mere surgical preference. With studies largely being relatively small retrospective case series with sometimes differing conclusions, it remains difficult to know when anterior pelvic ring fixation is imperative, helpful, or unnecessary. Future research should also be directed towards the establishment of specific pelvic fixation criteria in each pattern of sacral fracture, especially highly unstable injuries.

### 3.3 Reduction goals and treatment options

#### 3.3.1 Goals of treatment

The primary objective in treating sacral fractures is to optimize patient recovery through the least invasive methods. Management strategies include early mobilization and weight bearing, or fracture reduction and stabilization, and neural decompression when indicated. Treatment selection should be guided by both injury-specific factors and patient-related considerations, including sacral anatomy, associated injuries, medical comorbidities, functional status, and capacity to comply with weight-bearing limitations.

#### 3.3.2 Non-operative management

Non-operative management is appropriate for stable injuries where appropriate alignment is maintained. However, there are no established evidence-based guidelines regarding what constitutes “maintained alignment.” In practice, this treatment option is reserved for most low-energy incomplete or minimally displaced AOSpine Type B injuries, Type C0 sacral insufficiency fractures, and Type A injuries where pelvic ring stability is not compromised.^32^ Conversely, for most high-energy sacral fractures and for patients with persistent, function-limiting pain despite conservative care, operative stabilization is indicated.

#### 3.3.3 Operative management

Operative treatment is to be pursued in the setting of pelvic ring or lumbosacral instability, acute neural compression, or disabling pain. The primary goals of surgical intervention are to relieve neural compression, achieve fracture reduction, promote union, and prevent symptomatic deformity. Key considerations include surgical timing, soft tissue management in open injuries, instrumentation strategy (iliosacral fixation versus spinopelvic fixation), reduction techniques, and the need for decompression or lumbosacral fusion, and postoperative activity restrictions.

In unstable sacral injuries, surgical timing is determined less by fracture morphology and more by the patient's hemodynamic stability, neurologic status, and associated injuries. In the setting of resulting hemodynamic instability, initial interventions—including binder placement, embolization, pelvic packing, and/or external fixation—aim to effectively reduce intrapelvic volume and control bleeding.^33^ If definitive fixation must be delayed, temporizing measures such as longitudinal traction for vertically displaced fractures may facilitate later reduction.

In open injuries, prompt administration of broad-spectrum antibiotics and early debridement are imperative. Definitive fixation is often only pursued once soft tissue stability is achieved. Management of Morel-Lavallee lesions anecdotally varies from minimally affecting treatment to some providers staging instrumentation after an initial irrigation and debridement. Minimizing soft tissue disruption, closing fascial defects, and avoiding prominent instrumentation are key to reducing postoperative complications in the setting of high-energy injuries.^13^

##### 3.3.3.1 Sacral kyphosis – significance and reduction techniques

Posterior pelvic ring sagittal plane malreductions are associated with poor long-term clinical outcomes.^34^^,^^35^ In fractures with a transverse sacral component and associated kyphosis or translation as described by Roy-Camille, anatomic reduction restores alignment of the sacral canal and facilitates neurologic decompression. Residual sacral kyphosis acutely alters pelvic incidence, potentially resulting in symptomatic regional and global sagittal plane deformity if not corrected, as the lumbar spine will have to compensate to maintain sagittal balance.^36^

**Fig. 3:**
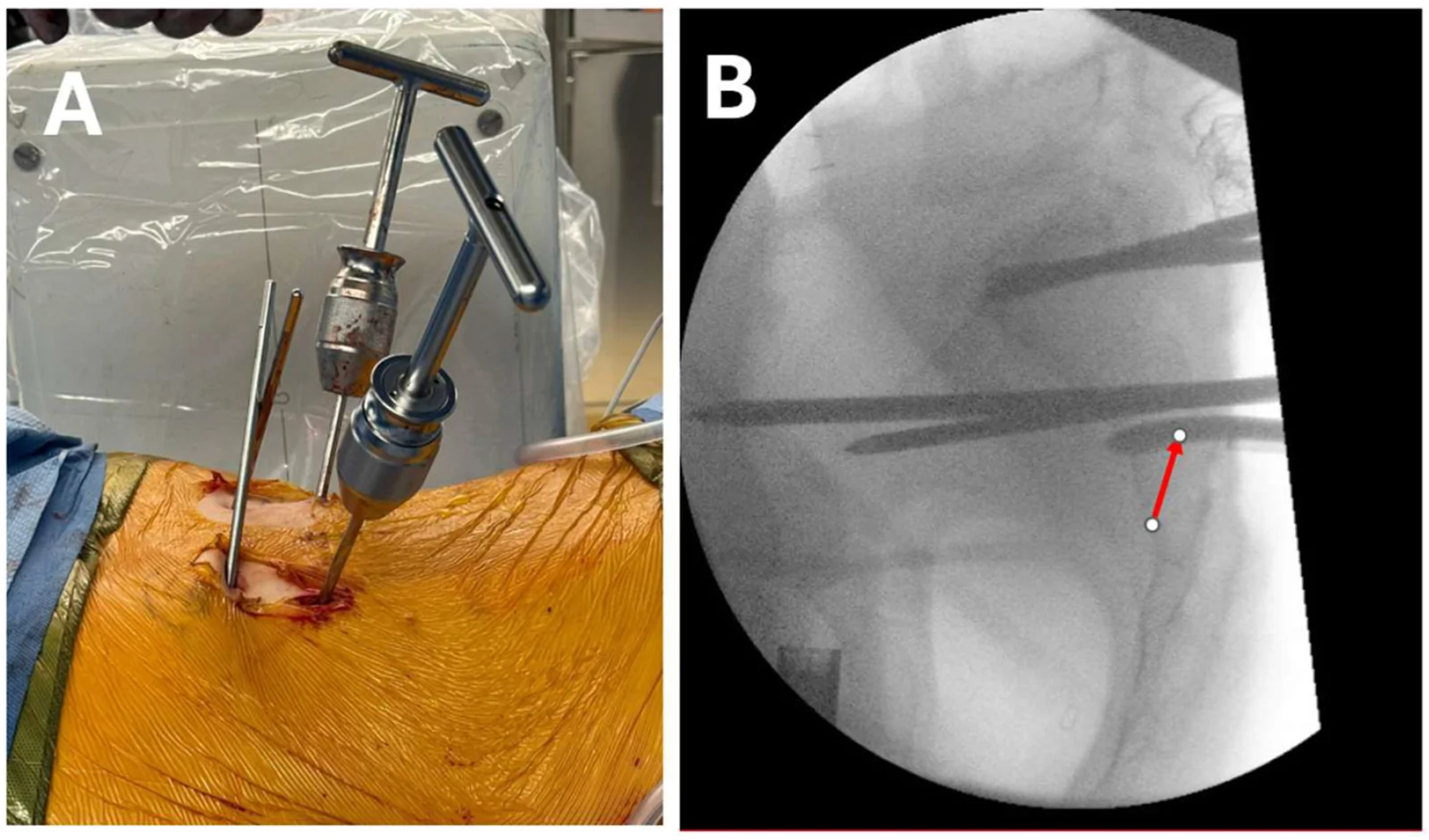
**Sacral kyphosis reduction**. (A) Case depicting percutaneous reduction of a sacral U type fracture. Bilateral Schantz pins with T-handles attached are percutaneously placed in the S1 pedicles. Bilateral pins are also placed in the ilia and direct manipulation allows for improvement in the sacral kyphosis. (B) Case where an open reduction was performed with intraoperative fluoroscopic image demonstrating a cobb elevator(red arrow) directly mobilizing the fracture after a sacral laminectomy was performed and thecal sac retracted.

Reduction follows a stepwise approach, progressing from closed to percutaneous, and finally to open techniques until an acceptable reduction is achieved (Fig. 3):

1. **Closed (Indirect) Reduction** – Initial attempts include skeletal traction, bump manipulation, or external fixation.
2. **Percutaneous Reduction** – If closed methods are inadequate, percutaneous techniques such as traction and provisional fixation may be employed. Successful reduction through closed or percutaneous means allows for definitive percutaneous fixation.^37^
3. **Open (Direct) Reduction** – Reserved for cases requiring direct neural decompression or when indirect methods fail. In complex injuries with displaced transverse components (e.g., AO Type C3 sacral-U variants), open techniques may involve sacral nerve root retraction, use of impactors, Schanz pins, and direct manipulation.

Despite the ability to reduce focal kyphosis with increasing surgical invasiveness and retrospective studies demonstrating worse outcomes with sacral malreductions, there is no agreed-upon amount of acceptable sacral kyphosis to guide when an escalation of surgical invasiveness, and associated complication profile, is in the best interest of the patient.^13^^,^^38^ However, normal spinal alignment parameters (pelvic incidence 30-80°, sacral slope 30-50°) may guide the adequacy of sacral kyphosis reduction, though these ranges are quite wide.^39^ Further, in pelvic ring injuries that involve both anterior and posterior components, reductions of these components often are approached in tandem, as malalignment in one region can compromise the reduction of the other. In cases requiring spinopelvic fixation, particularly with complex sacral injuries, coordinated management between providers with pelvic trauma and spine expertise is often valuable.

### 3.4 Instrumentation options

In operative injuries, the choice of instrumentation is guided by the injury pattern, patient anatomy, and expected post-operative biomechanical demands of the construction. While younger patients may tolerate weight-bearing restrictions, such limitations are often impractical in polytrauma or geriatric populations. Current fixation strategies primarily include percutaneous sacroiliac screws and spinopelvic fixation, as techniques such as direct sacral plating, trans-iliac bars, and posterior bridge plating have fallen out of favor due to high complication and failure rates.

#### 3.4.1 Percutaneous sacroiliac fixation

Percutaneous sacroiliac fixation involves the placement of cannulated screws into either the iliosacral corridor (oriented perpendicular to the SI joint) or the trans-iliac trans-sacral corridor (spanning both ilia and the sacrum) following fracture reduction.^40^ This technique remains the preferred method for most unstable AOSpine Type B injuries without significant vertical displacement, provided that suitable osseous corridors are present in the upper and second sacral segments to accommodate screw placement. Successful instrumentation depends on accurate fracture reduction, as malreduction narrows the osseous corridors.^41^ Preoperative CT imaging is essential for identifying sacral dysmorphism, which alters the anatomy of the S1 segment and may preclude safe trans-iliac trans-sacral screw placement. In such cases, CT-guided screw placement or standard sacroiliac screws should be used to avoid injury to the L5 nerve roots^42^^,^^43^

When sacroiliac screws are indicated, these screws can be placed in a manner (lag-by-design or lag-by-technique) to generate compression across parasagittal fracture lines and reduce the risk of nonunion. However, in vertically unstable injuries, sacroiliac screws alone may not sufficiently resist shear forces, warranting adjunctive fixation such as spinopelvic constructs or triangular osteosynthesis.^44^

#### 3.4.2 Spinopelvic fixation

Spinopelvic fixation entails posterior spine-based instrumentation from the lower lumbar spine or sacrum to the ilium. This technique provides greater biomechanical stability—especially in flexion-extension—and permits immediate weight bearing.^45^ Both open and percutaneous approaches are described, with open methods reserved for cases requiring direct fracture reduction.^13^^,^^37^ The proximal extent of instrumentation is often debated.

Spinopelvic fixation is typically indicated for vertically unstable AOSpine Type C1–C3 injuries and may be constructed unilaterally or bilaterally depending on the fracture pattern. Iliac hardware should be recessed below the fascia to minimize the risk of symptomatic prominence and wound complications. When the lumbosacral junction is spanned, fusion is considered in injuries where the lumbosacral facet is disrupted (e.g., C1 injuries). In cases where a fusion is not indicated, consideration is given to hardware removal after fracture union or expectant breakage of hardware.^13^ Though well described, there remains no consensus on specific aspects such as the proximal extent of construct (e.g., S1, L5 versus L4), when to consider unilateral versus bilateral instrumentation, or the utility of a cross-connector.

#### 3.4.3 Triangular osteosynthesis

Triangular osteosynthesis combines sacroiliac screws with spinopelvic fixation, enhancing vertical stability. The sacroiliac screws provide fracture compression across vertically oriented fracture lines, while the spinopelvic construct functions as a neutralization mechanism to counteract hemipelvis flexion and proximal migration. This technique has demonstrated biomechanical integrity and allows for early weight bearing.^46^

For complex injuries with both vertical and horizontal components (e.g., lambda- or H-type C3 fractures), a trans-iliac trans-sacral screw or cross connector may be added to bilateral spinopelvic fixation to prevent lateral migration of the pelvis and loss of reduction.

Though the principles that guide the choice of instrumentation option are well understood, there remain very few studies that compare clinical outcomes of different instrumentation choices and techniques (e.g., open versus percutaneous or fluoroscopic based versus navigation) in specific injury patterns. Further, nearly all existing studies present a retrospective case series using a specific technique and compare against a historical control, illustrating the need for prospective trials.

### 3.5 Decompression of nerve roots – indications and timing

Neurologic impairment is a well-described complication, especially in U or H-shaped sacral fractures with spinopelvic dissociation (AOSpine type C3) where the spinal canal is also involved.^47^ The severity of neurological injury varies from neuropathy to complete cauda equina syndrome or nerve root injury. Neurological examination of the patients may be challenging since they often have other severe injuries, and also because of the pain or disorientation.^48^ In addition to the standard lower extremity motor and sensory neurological examination, perineal sensation and rectal tone should be examined for prompt diagnosis of cauda equina syndrome.

In patients with neurologic impairment and ongoing neural element compression, the decompression of the spinal canal and exiting nerve roots may be performed by using either indirect or direct methods.^48^ In the indirect method, the reduction of the fracture also relieves compression of the cauda equina and nerve roots. If the adequate correction is not achieved or if there is residual nerve or cauda equina compression, direct decompression by laminectomy should be considered. The majority of the patients have been reported to have full or partial neurological recovery after fracture fixation and decompression.^21^

Since these high-energy trauma patients may have other life-threatening injuries, the definitive surgery is demanding, and the surgical treatment may delay. In the retrospective study of Ayoub^49^ the neurological recovery was better if the posterior decompression and fixation of the fracture were performed during the first 3 days. However, in another retrospective analysis of Schildhauer et al.^21^ there was no association between timing of decompression and neurological outcome. Instead, they found an association between the severity of initial presentation based on incomplete cauda equina syndrome and continuity of all sacral roots with the complete neurological recovery. The degree of initial transversal displacement in patients with spinopelvic dissociation has also been shown to be associated with neurological recovery.^50^

The studies investigating the need for decompression and timing of surgery in patients with high-energy sacral fractures with neurological deficit are sparse, small, and mainly retrospective. To better understand the significance of the surgical method and the urgency of the surgical treatment, there is a need for studies with more patients and a more precise design.

## 4 Limitations

There are several limitations to this study. The studies included in this narrative review are heterogeneous with regards to injury patterns, degrees of instability, and outcome parameters. The relative rarity of these injuries, combined with the lack of high-quality comparative studies, substantially limits the strength of the conclusions, particularly concerning treatment options. Furthermore, this review encompasses five distinct areas within the field of sacral fractures and is partly based on expert opinion. Therefore, the conclusions should be interpreted with caution.

## 5 Conclusion

Sacral fractures present in a highly heterogeneous manner and require many clinical decisions on the part of the treating surgeon. These include the need to determine an injury's mechanical stability by factoring in fracture morphology and the contribution of associated pelvic ring disruption, the need for neurologic decompression, and the need for stabilization. If stabilization is indicated, the optimal construct would facilitate bony union and early mobilization.

Despite advancements in the classification proposed by the AOSpine, which has a good reliability and a crescent degree of severity, management is still based on expert opinions and regional preferences, with limited evidence. For these reasons, further prospective, multicenter, and well-designed trials are strongly necessary to define better treatment algorithms and answer the raised knowledge gaps.

## Guardian/patient's consent

this article is a review of previously published literature and does not involve direct participation of patients or guardians; therefore, consent was not required.

## Ethical statement

this article is a review of previously published literature. No new studies involving human participants or animals were performed by the authors; therefore, ethical approval and informed consent were not required.

## Author credit statement

ANE: Conceptualization, Literature search, Methodology, Writing – original draft, review & editing, Supervision, Visualization.

MHN: Literature search, Data curation, Writing – original draft.

AAH: Conceptualization Literature search, Writing – original draft.

GDS: Formal analysis, Writing – review & editing.

MS: Formal analysis, Writing – review & editing.

SFB: Formal analysis, Writing – review & editing.

AFJ: Writing – review & editing.

KS: Writing – review & editing.

RB: Supervision, Writing – review & editing, Project administration.

EY: Conceptualization, Literature search, Writing – original draft, review & editing, Project administration.

## Funding statement

the authors disclosed receipt of the following financial support for the research, authorship, and/or publication of this article: This study was organized and funded by AO Spine through the AO Spine Knowledge Forum Trauma & Infection. Study support was provided directly through AO Innovation Translation Center, Network Clinical Research.

## Declaration of competing interest

The authors declare that they have no known competing financial interests or personal relationships that could have appeared to influence the work reported in this paper.

## References

[1] Santolini E., Kanakaris N.K., Giannoudis P.V. (May 2020). Sacral fractures: issues, challenges, solutions. EFORT Open Rev.

[2] Rodrigues-Pinto R., Kurd M.F., Schroeder G.D. (Oct 2017). Sacral fractures and associated injuries. Glob Spine J.

[3] Aprato A., Branca Vergano L., Casiraghi A. (Sep 4 2023). Consensus for management of sacral fractures: from the diagnosis to the treatment, with a focus on the role of decompression in sacral fractures. J Orthop Traumatol.

[4] Barber L.A., Katsuura Y., Qureshi S. (May 2023). Sacral fractures: a review. HSS J.

[5] Lee Y., Lambrechts M., Narayanan R. (Feb 1 2024). The surgical algorithm for the AO spine sacral injury classification system. Spine.

[6] Schroeder G.D., Kurd M.F., Kepler C.K. (Nov 2016). The development of a universally accepted sacral fracture classification: a survey of AOSpine and AOTrauma members. Glob Spine J.

[7] Kweh B.T.S., Tee J.W., Oner F.C. (Dec 1 2022). Evolution of the AO spine sacral and pelvic classification system: a systematic review. J Neurosurg Spine.

[8] Vaccaro A.R., Schroeder G.D., Divi S.N. (Aug 19 2020). Description and reliability of the AOSpine sacral classification system. J Bone Joint Surg Am.

[9] Denis F., Davis S., Comfort T. (Feb 1988). Sacral fractures: an important problem. Retrospective analysis of 236 cases. Clin Orthop Relat Res.

[10] Dreizin D., Smith E.B. (Nov-Dec 2022). CT of sacral fractures: classification systems and management. Radiographics.

[11] Wheeldon J.A., Pintar F.A., Knowles S., Yoganandan N. (2006). Experimental flexion/extension data corridors for validation of finite element models of the young, normal cervical spine. J Biomech.

[12] Zheng Y., Lu W.W., Zhu Q., Qin L., Zhong S., Leong J.C. (Feb 1 2000). Variation in bone mineral density of the sacrum in young adults and its significance for sacral fixation. Spine.

[13] Bellabarba C., Schildhauer T.A., Vaccaro A.R., Chapman J.R. (May 15 2006). Complications associated with surgical stabilization of high-grade sacral fracture dislocations with spino-pelvic instability. Spine.

[14] Roy-Camille R., Saillant G., Gagna G., Mazel C. (Nov 1985). Transverse fracture of the upper sacrum. Suicidal jumper's fracture. Spine.

[15] Vaccaro A.R., Kim D.H., Brodke D.S. (2004). Diagnosis and management of sacral spine fractures. Instr Course Lect.

[16] Beucler N. (Mar 24 2025). Introducing the relationship between pelvic incidence and subtype of U-shaped sacral fracture according to roy-Camille classification: how to restore sagittal balance in spinopelvic trauma. Injury.

[17] Isler B. (1990). Lumbosacral lesions associated with pelvic ring injuries. J Orthop Trauma.

[18] Kanna R.M., Pratheep K.G., Shetty A.P., Rajasekaran S. (Jan 2023). Do all Isler's type sacral fractures necessarily require surgical fixation?. Eur Spine J.

[19] Meissner-Haecker A., Diaz-Ledezma C., Klaber I. (Feb 2022). Inter- and intra-observer agreement using the new AOSpine sacral fracture classification, with a comparison between spine and pelvic trauma surgeons. Injury.

[20] Burgess A.R., Eastridge B.J., Young J.W. (Jul 1990). Pelvic ring disruptions: effective classification system and treatment protocols. J Trauma.

[21] Schildhauer T.A., Bellabarba C., Nork S.E. (2006). Decompression and lumbopelvic fixation for sacral fracture-dislocations with spino-pelvic dissociation. Article. J Orthop Trauma.

[22] Achor T.S., Alonso J.E., Arduini M. (2015). Fractures of the Pelvis and Acetabulum.

[23] Tile M. (May 1996). Acute pelvic fractures: I. Causation and classification. J Am Acad Orthop Surg.

[24] Sagi H.C., Ordway N.R., DiPasquale T. (Mar 2004). Biomechanical analysis of fixation for vertically unstable sacroiliac dislocations with iliosacral screws and symphyseal plating. J Orthop Trauma.

[25] Crist B.D., Pfeiffer F.M., Khazzam M.S., Kueny R.A., Della Rocca G.J., Carson W.L. (Jan 2019). Biomechanical evaluation of location and mode of failure in three screw fixations for a comminuted transforaminal sacral fracture model. J Orthop Translat.

[26] Bodzay T., Sztrinkai G., Pajor S. (2014). Does surgically fixation of pubic fracture increase the stability of the operated posterior pelvis?. Eklem Hastalik Cerrahisi.

[27] Tucker N.J., Scott B.L., Heare A., Stacey S.C., Mauffrey C., Parry J.A. (Apr 1 2023). Combined anterior-posterior versus posterior-only fixation of stress-positive minimally displaced lateral compression type 1 (LC1) pelvic ring injuries. J Orthop Trauma.

[28] Höch A., Schneider I., Todd J., Josten C., Böhme J. (2018/04/01 2018). Lateral compression type B 2-1 pelvic ring fractures in young patients do not require surgery. Eur J Trauma Emerg Surg.

[29] Avilucea F.R., Archdeacon M.T., Collinge C.A., Sciadini M., Sagi H.C., Mir H.R. (Sep 5 2018). Fixation strategy using sequential intraoperative examination under anesthesia for unstable lateral compression pelvic ring injuries reliably predicts union with minimal displacement. J Bone Joint Surg Am.

[30] Aggarwal S., Patel S., Mehta L., Kataria M., Kumar V., Kumar P. (Jul 3 2024). Posterior-only fixation in pelvic fractures: is it sufficient in lateral compression injuries?. Chin J Traumatol (Engl Ed).

[31] Moussa I.S., Sallam A.M., Mahmoud A.K., Elzaher E.H., Nagy A.M., Eid A.S. (Jan 2023). Combined anterior and posterior ring fixation versus posterior ring fixation alone in the management of unstable Tile B and C pelvic ring injuries: a randomized controlled trial. Chin J Traumatol (Engl Ed).

[32] Meinberg E.G., Agel J., Roberts C.S., Karam M.D., Kellam J.F. (Jan 2018). Fracture and dislocation classification Compendium-2018. J Orthop Trauma.

[33] Marmor M., El Naga A.N., Barker J., Matz J., Stergiadou S., Miclau T. (2020). Management of pelvic ring injury patients with hemodynamic instability. Front Surg.

[34] Lee H.D., Jeon C.H., Won S.H., Chung N.S. (Jul 2017). Global sagittal imbalance due to change in pelvic incidence after traumatic spinopelvic dissociation. J Orthop Trauma.

[35] Lai C.Y., Lai P.J., Tseng I.C. (Mar 2022). Postoperative reduction quality may be the Most important factor that causes worse functional outcomes in open and closed pelvic fractures. World J Surg.

[36] Hart R.A., Badra M.I., Madala A., Yoo J.U. (Jul 2007). Use of pelvic incidence as a guide to reduction of H-type spino-pelvic dissociation injuries. J Orthop Trauma.

[37] Khela M., Agha O., Bonsignore-Opp L., Xu M., Gendelberg D., El Naga A.N. (Mar 24 2025). Percutaneous spinopelvic fixation technique using external fixation for focal kyphosis reduction in U-type sacral fractures: a case report. J Spine Surg.

[38] Nork S.E., Jones C.B., Harding S.P., Mirza S.K., Routt M.L. (May 2001). Percutaneous stabilization of U-shaped sacral fractures using iliosacral screws: technique and early results. J Orthop Trauma.

[39] Cho Y., Jo D.J., Hyun S.J., Park J.H., Yang N.R. (Jun 2023). From the spinopelvic parameters to global alignment and proportion scores in adult spinal deformity. Neurospine.

[40] Bishop J.A., Routt M.L. (Jun 2012). Osseous fixation pathways in pelvic and acetabular fracture surgery: osteology, radiology, and clinical applications. J Trauma Acute Care Surg.

[41] Routt M.L., Simonian P.T., Agnew S.G., Mann F.A. (1996). Radiographic recognition of the sacral alar slope for optimal placement of iliosacral screws: a cadaveric and clinical study. J Orthop Trauma.

[42] Miller A.N., Routt M.L. (Jan 2012). Variations in sacral morphology and implications for iliosacral screw fixation. J Am Acad Orthop Surg.

[43] Falzarano G., Rollo G., Bisaccia M. (2018). Percutaneous screws CT guided to fix sacroiliac joint in tile C pelvic injury. Outcomes at 5 years of follow-up. SICOT J.

[44] Sagi H.C., Militano U., Caron T., Lindvall E. (May-Jun 2009). A comprehensive analysis with minimum 1-year follow-up of vertically unstable transforaminal sacral fractures treated with triangular osteosynthesis. J Orthop Trauma.

[45] Acklin Y.P., Zderic I., Richards R.G., Schmitz P., Gueorguiev B., Grechenig S. (Jun 2018). Biomechanical investigation of four different fixation techniques in sacrum denis type II fracture with low bone mineral density. J Orthop Res.

[46] Schildhauer T.A., Ledoux W.R., Chapman J.R., Henley M.B., Tencer A.F., Routt M.L. (Jan 2003). Triangular osteosynthesis and iliosacral screw fixation for unstable sacral fractures: a cadaveric and biomechanical evaluation under cyclic loads. J Orthop Trauma.

[47] Konig M.A., Jehan S., Boszczyk A.A., Boszczyk B.M. (May 2012). Surgical management of U-shaped sacral fractures: a systematic review of current treatment strategies. Eur Spine J.

[48] Williams S.K., Quinnan S.M. (Sep 2016). Percutaneous lumbopelvic fixation for reduction and stabilization of sacral fractures with spinopelvic dissociation patterns. J Orthop Trauma.

[49] Ayoub M.A. (Sep 2012). Displaced spinopelvic dissociation with sacral cauda equina syndrome: outcome of surgical decompression with a preliminary management algorithm. Eur Spine J.

[50] Lindahl J., Makinen T.J., Koskinen S.K., Soderlund T. (Dec 2014). Factors associated with outcome of spinopelvic dissociation treated with lumbopelvic fixation. Injury.

